# Optimal Performance and Modeling of Wireless Technology Enabling Smart Electric Metering Systems Including Microgrids

**DOI:** 10.3390/s21217208

**Published:** 2021-10-29

**Authors:** Carlos Suárez, Esteban Inga

**Affiliations:** 1 Master’s Program in Electricity, Universidad Politécnica Salesiana, Quito EC170525, Ecuador; csuarezp@est.ups.edu.ec; 2 Postgraduate Department, Smart Grid Research Group (GIREI), Universidad Politécnica Salesiana, Quito EC170525, Ecuador

**Keywords:** geo-referencing, heuristic techniques, smart metering, micro grids, optimization, set cover problem

## Abstract

This work is focused on the performance analysis and optimal routing of wireless technology for intelligent energy metering, considering the inclusion of micro grids. For the study, a geo-referenced scenario has been taken into account, which will form the structure of a graph to be solved using heuristic-based algorithms. In the first instance, the candidate site of the world geography to perform the case study is established, followed by deploying infrastructure devices and determining variables and parameters. Then, the model configuration is programmed, taking into account that a set of nodes and vertices is established for proper routing, resulting in a preliminary wireless network topology. Finally, from a set of restrictions, a determination of users connected to the concentrator and optimal routing is performed. This procedure is treated as a coverage set problem. Consequently, to establish the network parameters, two restrictions are specifically considered, capacity and range; thus, can be determined the best technology to adapt to the location. Finally, a verification of the resulting network topologies and the performance of the infrastructure is done by simulating the wireless network. With the model created, scenarios are tested, and it is verified that the optimization model demonstrates its effectiveness.

## 1. Introduction

The current scenario of electric grids is focused on the deployment of advanced devices, the use of techniques and tools for system control and monitoring, in addition to the management of data that are the product of this process and the active participation of customers in the electricity market; in this way, a characteristic infrastructure of the smart grid is configured to frame a safe, reliable and quality operation [1,2,3,4]. In addition, the grid configurations have been delineated to adopt distributed generation (DG) and renewable energies. Therefore, micro grids and nano grids are currently managed, which have similar characteristics to conventional power grids, but of reduced dimension [2,5]; through these, a bidirectional electricity market can be developed [5,6], which on the one hand provides certain advantages in terms of DG adaptation, but on the other hand generates disadvantages concerning control, regulation, protection and customer participation [7,8]. Undoubtedly, the key to the future of smart grids is aimed at the conformation of smart micro grids and the inclusion of advanced control techniques for their interaction with conventional grids.

Given the reason mentioned above, research has been developed to solve the problem that currently governs communications, which becomes complex due to its large number of variables and restrictions [9,10]. The problems related to chargeability variations, reactive compensation, inertial behavior, among others that define the characteristics of the current electrical grid, are modified in particular by the increase of renewable energies and their dynamic behaviors within the grid; for this reason, proposals and innovations have been developed through techniques that seek to solve the difficulties of the electrical phenomenon [11]. However, other problems have to do with the treatment and management of the data obtained from the advanced communication system proper to the smart grid. Therefore, it is necessary to mention that, within the current dilemmas to be solved, an adequate deployment of the infrastructure supports bidirectional communication between the system’s actors.

In deploying a smart grid, the configuration of a micro grid (MG) becomes an indispensable consideration for the integration of distributed generation units; since its adequacy allows interaction with the conventional grid of the interconnected system. Advanced metering infrastructure (AMI) micro grids can improve electric service efficiency and contribute to moderate energy consumption [12,13]. According to Inga et al. in their 2017 paper [14,15], for AMI to be considered an efficient and reliable component of the smart grid (SG), it is necessary to ensure two-way communication between the smart meter (SM) and the electric utility. According to [1,2,16], the key in SG is the integration of an adequate communication infrastructure that allows monitoring and gives way to effective control of the electric grid; in addition, it should be taken into account that the data obtained from AMI are necessary for power quality management, especially if we integrate electric micro grids [7,8,11]; additionally, advanced infrastructures allow metering data to be managed for MG operation, and load forecasting to the system [17,18].

The problem concerning communications utilizes optimization techniques; a more concise approach to the infrastructure deployment solution can be given, explicitly considering using wireless communication devices under different technologies and standards. In addition, it warns the need to test the performance of the wireless network in the processing of data coming from micro grids and conventional clients [7].

This article is organized as follows. Section 2 describes related work. Section 3 describes the problem formulation. In Section 4, the analysis of simulation results is carried out. Finally, we conclude this paper in Section 5.

## 2. Related Works

In compliance with the standards of quality, safety and reliability of the electrical infrastructure, it is imperative to have a communication system that allows meeting the transactional needs of supply and demand [9], also considering that the electrical phenomena of the network in the face of changes in chargeability or availability of generation may occur due to the inclusion of renewable energies and electric cars [19].

As indicated by the study of [11], the most appropriate available means of communication should be used for the interconnection of the network elements since the bidirectional communication required implies a reasonably robust network for the handling of measured data. Consequently, in previous studies in smart metering, there are two types of control architectures for the MG [20,21,22]. For such reason, it can differentiate the centralized architectures, where a central MG controller (CCMG) agglomerates the data for further processing and decision making; on the other hand, if we consider a decentralized system, each DG unit (distributed generation) and controllable load has its controller; to subsequently manage actions based on predefined strategies. If we consider a centralized scheme, the CCMG controls the information exchanges at three levels: distributed generation DG, Grid and Load; these levels consider aspects related to the customer’s load profile, intermittent sources and electricity markets.

**Figure 1:**
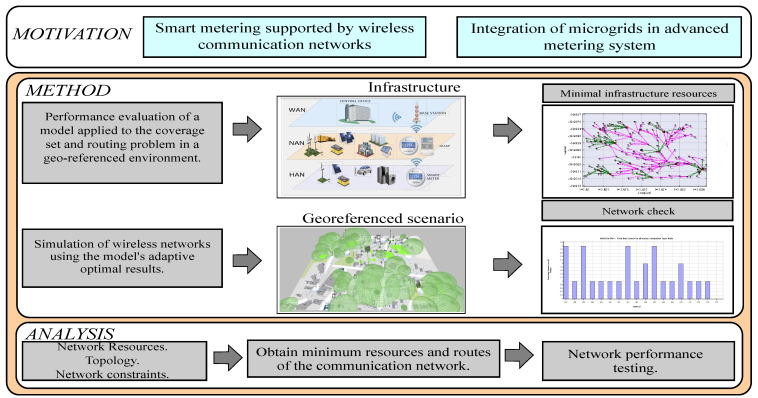
Conceptual graph of the optimal performance of a wireless network infrastructure.

Three types of areas can be identified in communication infrastructures. The first is HAN, Home Area Network, which represents the first section of the communications process, as shown in Figure 1, which encompasses communications by the interaction of intelligent devices within the home, the SM and the UDAP in a configuration is usually star type. However, optimization functions can consider P2P (point to point) or meshed networks.

The bidirectional communication of the elements of this is necessary for control and monitoring, making use of wired or wireless technology to exchange information of power, voltage, current and frequency measurements. The information collected by the SM should be treated according to the residential, commercial or industrial origin. NAN, Neighborhood Area Network, represents the intermediate section of the smart metering communications process, as shown in Figure 1. Within the micro-electrical grid, this network aims to form the links between the UDAP and the CCMG. In this area, bidirectional communication must be ensured to transfer information from the customers to the micro grid controller and vice versa, using the UDAP [20]. Wide Area Network (WAN), represents the upper section of the smart metering communication process as seen in Figure 1. This network is characterized by handling long distances of the communication links between central offices of the network with the different power substations and the interaction between the public power grid and the micro grid by using base stations for data transmission. This network, also known as an access area network, allows the external exchange of information between the distribution network operator, market operator, and other CCMG.

**Figure 2:**
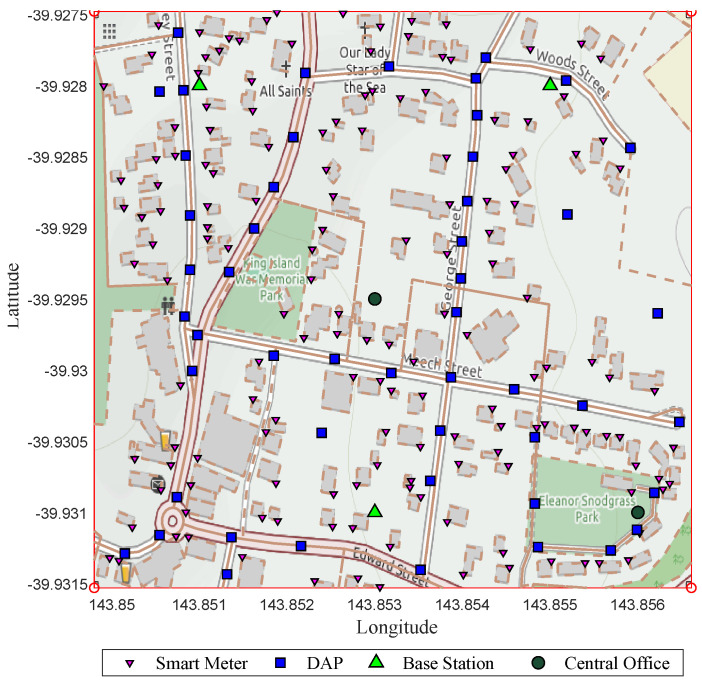
Geo-referenced case study and its infrastructure elements.

The concept of heterogeneous networks contemplated in previous studies establishes that using different technologies in the transfer of information from the AMI allows reducing costs, ensuring that the connectivity required by the service between customers, neighboring micro grids and the distribution or marketing company within the concession area is not compromised. Consequently, also warns that it is necessary to consider the nature of the technologies that can be chosen as solutions; since network topologies, communication protocols, security, interference, among other aspects are determinant for the optimal performance of the network. Following this line of work, cellular technology can be considered an option for transferring last-mile information between the universal data aggregation points (UDAP) and the base stations (BS). In contrast, to transfer information from the SM to the UDAP they would work in a different wireless technology within the 900 MHz, 2.4 GHz or 5 GHz frequency bands, depending on the geographical location and availability of devices by the manufacturing companies. As shown in Figure 2, the metering infrastructure that includes micro grids contemplates a central office (CO) or those available in the scenario, a base station or those available in the area, a minimum number of UDAP and the smart meters that are available in the area for each customer. While in HAN the smart meters conglomerate the user’s consumption data, in NAN the conformation of links between the meters and the UDAP is adapted, and in WAN the exchange of information between the conventional energy marketer, the market operators, nearby micro grids and the users through the cellular communications base stations takes place. Smart meters and UDAP can use a meshed network topology and generate hops between them to find the route to their destination. However, a star topology can be examined as an option to configure the heterogeneous network, taking into account that for any feasible option, the UDAP would have double technology, one to connect with the SM and the other to connect with the nearby BS.

Micro grid communications are based on smart metering communications architectures where different wired and wireless technologies can be used. According to [20], there is a communications need for each type of network. In such effect, it performs a summary of the communication needs of the conformed area networks. In Table 1 shows the data speed, coverage range, bandwidth, latency and data traffic required for each of the system’s networks.

Because of the communication needs, the study of [14,23] considers an investigation in the function of heterogeneous wireless networks as a solution to communication in advanced measurement systems. Additionally, in [11,16] a model in function of combined networks between the optical fiber and wireless technologies can be evidenced. If working with wireless networks, according to [20,24,25], there is a classification of technologies according to the signal range distances for each area network, so the minimum requirements studies are limited to what device manufacturers can offer. In general, standards can be defined for the parameters offered by the technologies; in this sense, the available options are summarized in Table 2 in order to determine which ones can be applied in the geo-referenced environment.

## 3. Original Contribution

The present work proposes as innovation and novelty compared to the proposed works a scalable model based on graph theory considering the increase of sensors; additionally, the achieved model is contrasted in a simulation process that involves variables that the optimization model does not incorporate in its objective function. In this way, planning and deployment of wireless sensors incorporate reliability in the topology to be deployed. Usually, the modeling and simulation stages are treated independently, a situation articulated in a complementary manner in this proposal. Additionally, showing the result in a geo-referenced scenario involves an added value of importance to be used in investment proposals in different applications of smart cities or smart grids. Indeed, the cost of each UDAP depends on each wireless technology, being the highest cost the solution that involves a single technology such as cellular. The optimization model considers the number of UDAPs that in the original model is reduced, which shows that there is cost minimization. Since the model is flexible, it can involve several coverage radii depending on each wireless technology. It means that the cost of the model will be the final number of UDAP for the cost of the evaluated technology.

## 4. Problem Formulation

The data that would produce the communication between micro grids and consumer customers in the network depends on a single concentrator. It would generate high management of information as a centralized unit, which would give way to a problem of data traffic difficult to handle due to the necessary communication links between customers, the distribution company [20], and the micro grids of the place. For this reason, a decentralized architecture can be considered to allow distribution of metered data by coverage areas through grouping and hopping across concentrators and SM to be able to communicate with the CO using the minor link cost BS.

Depending on the type of consumer or customer, the micro grid model can be differentiated into commercial, home or industrial areas, from which the network bandwidth involving communication between the SM and the UDAP can be determined. In the network, bidirectional communication occurs between the CCMG, the concentrators, and the SM to transfer the measured information for its management and data processing. The WAN network is in charge of conglomerating the data for the management, among other micro grids, the adequacy to the requirements of the electricity market operator (MO) and the instructions of the distribution network operator (DNO).

In general terms, we would talk about a model that allows obtaining optimal results in deploying infrastructure to integrate micro grids, considering the minimization of data concentrator equipment, restricting the number of smart meters per concentrator and limiting the number of hops per link. For the model, it is necessary to perform minimum spanning trees, where the system distances are considered to define which links are the most feasible for communication between actors. A set cover problem must also be considered since a minimum set must be defined for all customers with their respective meters. Since the problem contemplates a geo-referenced scenario, it is necessary to consider the determination of distances between points on a sphere using the Haversine formula; therefore, the longitude and latitude data of each map element are needed.

The candidate site for analysis is a geographic portion of the rural population of King Island, located in Tasmania, Australia. As it is a place with the potential to insert isolated micro-grids, it was decided to carry out the study corresponding to its communications network. It should be noted that this site has developed a renewable energy integration project called KIREIP, which reduces greenhouse gas emissions and improves the stability and reliability of the electrical system. Due to its particular isolation, it is clear that it cannot be connected to Australia’s primary power grid, making it an ideal candidate to study possible smart metering scenarios. A small central portion of the site has been taken due to a higher density of concentrated customers compared to other areas where there is not a sufficient number of users to perform the performance study, in addition to the fact that the portion of land chosen allows an adequate visualization for the analysis of the deployment of devices. Under the above, it has been decided to establish 156 consumption measurement points, 50 data concentrators, three base stations and two central offices, giving 211 nodes connected. Figure 2 shows the distribution of infrastructure elements for the proposed heterogeneous wireless network.

A communication system solution for energy metering generates a set of nodes and links. Therefore, we consider a graph from which the sets of vertices and edges are established. Given this background, the model used in this study contemplates a Set Cover Problem (SCP), thus determining the application of an algorithm based on heuristics due to its efficiency and simplicity concerning computational time [26]. This problem has previously defined a topology created by the Prim [27] algorithm, which determines the minimum spanning tree considering a connection capacity constraint per node and a maximum range given in kilometers. Any positive integer can be included in the capacity variable, depending on the wireless technology, while the range is any number, element of positive rationals, and is also defined by the connection technology of the device.

In addition, to determine the distances between nodes of the system, the Haversine formula generates the graph’s edges, resulting in the conformation of the logical matrices necessary for the SCP, PRIM, and Dijkstra algorithms.

In this work model, the Dijkstra algorithm is considered to determine the shortest route to perform the routing between devices.

The computational cost described as an NP-Complete for MST problem warns as notation Big Oh—O(nlogn) and the set cover algorithm $log_{2}\left(n\right)/2$.

The names of each of the variables and parameters of the system and algorithms are established in Table 3. In addition, to test the model used, two stages are considered to establish a difference between a pre-established scenario and its optimal reconstruction. The first stage begins with a distance measurement through Haversine’s formula, taking into account that all devices must be adequately defined by their longitude and latitude, followed by the routing between all nodes through Dijkstra and calculating the total cost of the paths found. In this way, a tree or preliminary topology of the wireless network of the scenario can be obtained at the lowest cost, considering the shortest distance according to the pre-established range in meters for each node. This first stage works specifically as indicated by Algorithm 1, which is divided into three steps: the establishment of input data, the calculation of distances, and the determination of the minimum paths with their cost.
**Algorithm 1** Shortest Routes in a Wireless Network**Step: 1** Inputdata:$X_{u},Y_{u}\leftarrow \mathrm{Meter}\;\mathrm{coordinates}$$X_{d},Y_{d}\leftarrow \mathrm{UDAP}\;\mathrm{coordinates}$$X_{eb},Y_{eb}\leftarrow \mathrm{Base}\;\mathrm{station}\;\mathrm{coordinates}$$X_{c},Y_{c}\leftarrow \mathrm{Head}\;\mathrm{Office}\;\mathrm{Coordinates}$$N=card\left(X_{u}\right);\;M=card\left(X_{d}\right)$$K=card\left(X_{eb}\right);\;P=card\left(X_{c}\right)$$X_{T}=\left[X_{u},X_{d},X_{eb},X_{c}\right];\;Y_{T}=\left[Y_{u},Y_{d},Y_{eb},Y_{c}\right]$**Step: 2** Calculationofdistances:**for** $i=1:card(X_{T})$ **for** $j=1:card(Y_{T})$  $D\left(i,j\right)\leftarrow Haversine(\left[Y_{i}\;X_{i}\right],\left[Y_{j}\;X_{j}\right])$ **endfor****endfor**D(D=0)←∞$\alpha =N+M+K+P;\;D_{max}=\delta \;\forall \;\delta \in Q^{+}$$G_{\alpha ,\alpha }\leftarrow \mathrm{ceros};\;G\left(D\leq D_{max}\right)\leftarrow \mathrm{unos}$**Step: 3** Minimumroadsandroutecosts:[rpciai]←compresssparsematrix(G)A=[rpciai]pred←Dijkstra(A,α)CostT←RouteCost(α,pred)

In the second stage of the model, a new capacity constraint is added for the nodes and their respective scope to establish the actual condition of a device to establish a simultaneous connection with other devices in the network. As shown in Algorithm 2, the determination of the topology is made using Prim’s algorithm, from which some devices connected and not connected to the UDAP is obtained, with which we proceed to discard those meters not connected from the set of initial users. With the number of meters defined, the SCP is solved using the Greedy SCP algorithm, referred to as the Chvátal heuristic, from which the concentrators chosen for further processing are obtained. Having redefined the number of nodes in the system, the proposed model recalculates the distances through Haversine to form a new graph.

In addition, the model proposes the assignment of weights to establish the routing criteria of the devices, so the Algorithm 3 is established within the model to restrict and enforce the connection between devices in NAN and WAN.

Finally, through Dijkstra again, the shortest path between the nodes of the system is created to determine the spanning tree that is established as the final topology resulting from the heterogeneous wireless network.
**Algorithm 2** Optimal Number of UDAP in a Wireless Network**Step: 1** Inputdata:InitializevariablesCapNodo=μ∀μ∈N$Coverage=\lambda \;\forall \;\lambda \in Q^{+}$**Step: 2** Minimumspanningtreegeneration:$B_{M,N}\leftarrow zeros$**for** i=1:M $XY1=[X_{T}\left(1:N\right),Y_{T}\left(1:N\right)]$ $XY2=[X_{T}\left(N+i\right),Y_{T}\left(N+i\right)]$ tmp←Prim(XY1,XY2,CapNodo,Coverage) B(i,temp)←ones**endfor****Step: 3** SCPresolutionandUDAPselection:s←sum(B);L=1:Nind←find(s=0)$L\left(ind\right)\leftarrow ⌀;\;B\left(:,ind\right)\leftarrow ⌀;\;B=B^{\prime }$[solC,solL]←GreedySCP(B)$R\leftarrow ordered(solL^{\prime }{,}^{\prime }rows^{\prime })$$X_{el}=X_{d}\left(R\right);Y_{el}=Y_{d}\left(R\right)$$X_{d}=X_{el};Y_{d}=Y_{el}$**Step: 4** Calculationofdistances:$G\leftarrow ⌀;\;M=length(X_{el})\;;D\leftarrow ⌀$$X_{T}=\left[X_{u},X_{d},X_{eb},X_{c}\right];\;Y_{T}=\left[Y_{u},Y_{d},Y_{eb},Y_{c}\right]$**for** $i=1:card(X_{T})$ **for** $j=1:card(Y_{T})$  $D\left(i,j\right)\leftarrow Haversine(\left[Y_{i}\;X_{i}\right],\left[Y_{j}\;X_{j}\right])$ **endfor****endfor**D(D=0)←∞**Step: 5** Minimumroadsandroutecosts:$D_{max}\leftarrow \delta \;\forall \;\delta \in Q^{+}$$G\leftarrow Assignweights(D,D_{max},N,M,K,P)$[rpciai]←compresssparsematrix(G)A=[rpciai]pred←Dijkstra(A,α)CostT←RouteCost(α,pred)

**Algorithm 3** Assign Weights
**Input:** $D,D_{max},N,M,K,P$
**Output:** *G*
**Step: 1** 

Inputdata:



α=N+M+K+P



$G_{\alpha ,\alpha }\leftarrow \mathrm{zeros}$



$G(D\leq D_{max})\leftarrow \mathrm{ones}$



$G2_{\alpha ,\alpha }\leftarrow \mathrm{zeros}$



v2≈v1;v3>v9;v4≈v2;v5>v4



v6>>v3;v7>v5;v8≈v7;v9>v8



∀v1,v2,v3,v4,v5,v6,v7,v8,v9∈N

**Step: 2** 

Assignmentofweights:



G2(1:N,1:N)=v1



G2(1:N,N+1:N+M)=v2



G2(N+1:N+M,1:N)=v2



G2(1:N,N+M+1:N+M+K)=v3



G2(N+M+1:N+M+K,1:N)=v3



G2(1:N,N+M+K+1:N+M+K+P)=∞



G2(N+M+K+1:N+M+K+P,1:N)=∞



G2(N+1:N+M,N+1:N+M)=v4



G2(N+1:N+M,N+M+1:N+M+K)=v5



G2(N+M+1:N+M+K,N+1:N+M)=v5



G2(N+1:N+M,N+M+K+1:N+M+K+P)=v6



G2(N+M+K+1:N+M+K+P,N+1:N+M)=v6



G2(N+M+1:N+M+K,N+M+1:N+M+K)=v7



G2(N+M+1:N+M+K,N+M+K+1:N+M+K+P)=v8



G2(N+M+K+1:N+M+K+P,N+M+1:N+M+K)=v8



G2(N+M+K+1:N+M+K+P,N+M+K+1:N+M+K+P)=v9



G=G∗G2




## 5. Analysis of the Results

As mentioned in the formulation of the problem, the model contemplates two restrictions: capacity and range. Capacity establishes the number of devices that each node can conglomerate, while the range establishes the length in meters that the nodes have as a distance limit to connect.

In addition, to determine the performance of the model, six scenarios are proposed according to the ranges of 50, 75, 100, 200, 300, and 1000 m that can be tested at the candidate site; in each scenario, six finite numbers of capacity are tested, generating 36 results that will allow analyzing and determining the optimal solution for the site. From the allocation of weights, it is established that the smart meters have the possibility of connecting to generate multi hops or connect with the UDAP to establish communication with the data applicant or otherwise to receive data from the CO. On the other hand, the UDAP are required to connect with the meters and generate multi hops, which implies that their first wireless technology should consider mesh connections; the UDAP should also connect with the BS through cellular technology in their last mile. Therefore, the result is a concentrator with double technology to establish links between the supply and demand entities of the electric system. Therefore, the model has been restricted to 4 hops, and the weights for the construction of the MST have been detailed in the Algorithm 3.

It is also established that the UDAP cannot connect directly with the central offices since the base station is the one in charge of communicating them through the Internet, so its weight will be assigned so that the slogan is fulfilled. It is also necessary to mention that based on performance studies conducted by other researchers, a large capacity in terms of devices per node requires more time to achieve an adequate transmission probability, in addition to working with the specifications guaranteed by the manufacturers, so for this study, it is established capacities of 5, 10, 15, 20, 25 and up to 30 users per node to generate the tests.

**Figure 3:**
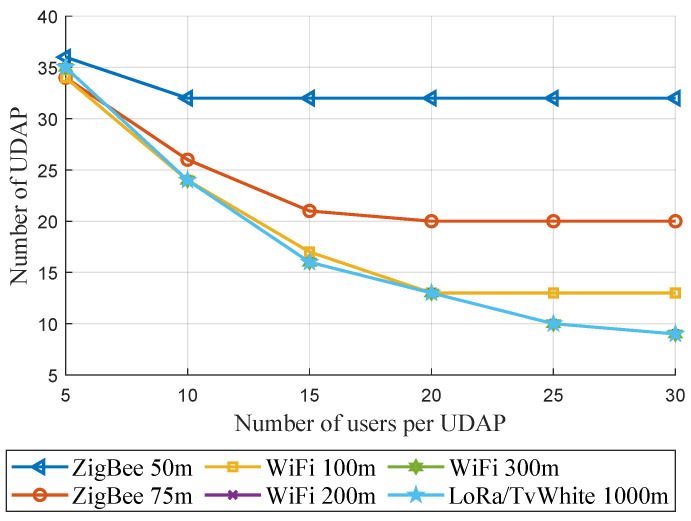
Number of users per UDAP vs. Number of UDAP.

The result of the number of users per concentrator concerning the optimal number of UDAPs can be seen in Figure 3, where it can be observed that the minimum number of optimized concentrators is 9, taking into account that the initial number was 50 and that for this result each UDAP can host up to 30 devices. Of the six ranges defined above, the results with range parameters greater than or equal to 200 m do not show changes. Therefore the program confirms that even if the range length increases, whatever the capacity is, the solution becomes invariant. It is verified that the capacity restricts the problem. However, for the candidate site and the number of devices established beyond a specific range, it is impossible to obtain better results. Similarly, it can be seen that in the scenario with a range parameter of 50 m, the minimum number of concentrators is not reduced to less than 32 UDAPs, even if the capacity is modified, which also makes it clear that in this case, the solution is invariant from a capacity of 10 users per node. This graph also shows that the optimization model. In addition, the minimization of the UDAPs is evident for the constraints established in each of the 36 trials performed.

**Figure 4:**
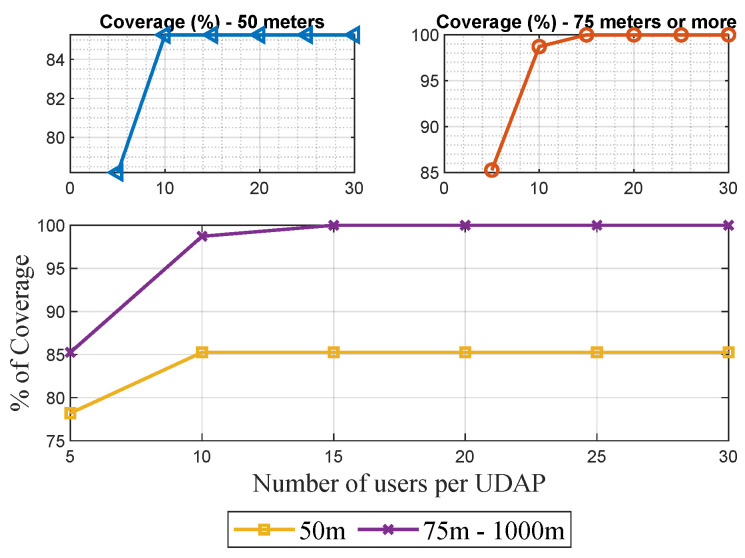
Percentage of devices covered—Greedy SCP.

In the Figure 4, is verified that in the proposed candidate site, Greedy SCP shows that with ranges of 50 m, it is not possible to connect all the devices in the network, while from 75 m of range, it is possible to ensure coverage of all the meters if the capacity restriction is greater than or equal to 15.

As a result, a range of 50 m or less is applied as a restriction; it will be challenging to establish routes with some devices. So it must be found that some nodes will be outside the tree built for the topology of the network.

The number of concentrators is reduced, and the model guarantees the construction of a tree with all network devices. It is necessary to establish a capacity parameter greater than or equal to 15 users and a reach greater than or equal to 75 m.

**Figure 5:**
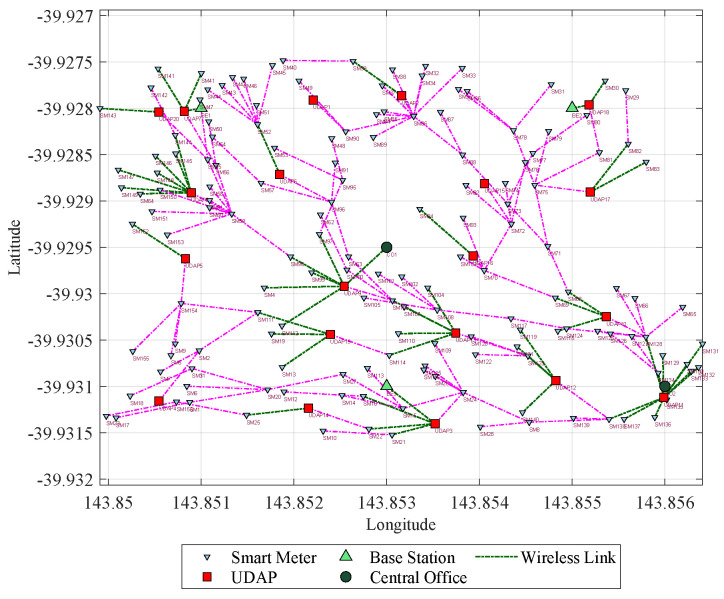
Topology with capacity of 20 users per node and range of 75 m.

**Figure 6:**
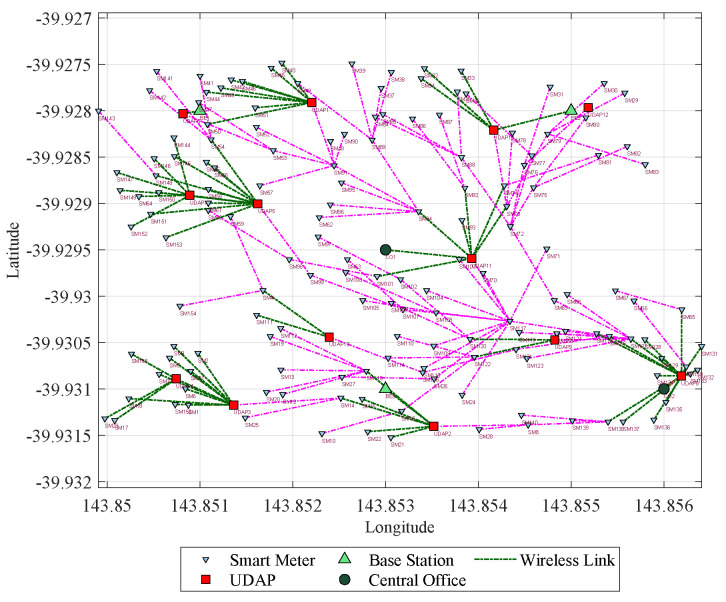
Final network topology of Scenario 4 with capacity of 20 users.

From the geo-referenced nodes, the result of the final topology considering Prim and SCP is presented Figure 5 shows the result of a scenario whose capacity restrictions are 20 devices per node and a range of 75 m; here, it can be evidenced the topology with 174 nodes and the routes that have been formed. In addition, it also shows that out of 50 UDAPs deployed, 30 have been reduced. On the other hand, Figure 6, shows the result of another scenario, whose range is restricted to 100 m and capacity to 20 users per node; here, it can be seen that from the 50 UDAPs of the proposed case, 37 are eliminated, and with the resulting 13 a new routing with the other elements of the network is generated. Therefore the applied model confirms its performance in optimizing infrastructure resources.

**Figure 7:**
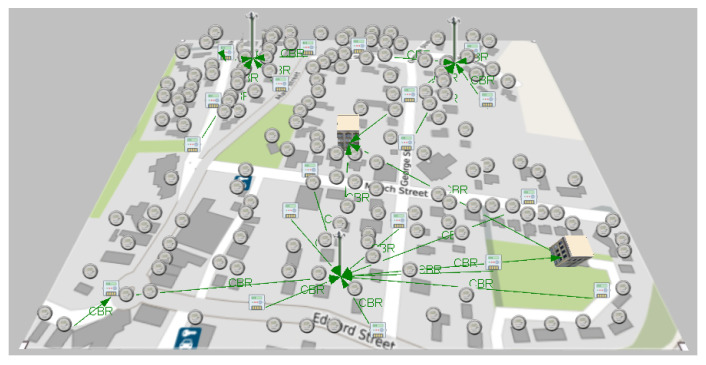
Zigbee network topology for simulation.

Employing the wireless network simulator QualNet Developer 5.2, the topology of the Zigbee network of Figure 7 obtained from the Matlab optimization algorithm is implemented, on the other hand, another WiFi scenario is configured with the same number of nodes, but with other configuration parameters; for both cases, the geographical location of the devices in the respective canvas is considered. A difference between the two technologies to be tested in WiFi and 19 local networks has been configured; conversely, in Zigbee, a single mesh network is used between the UDAPs and SMs of the infrastructure. Table 4 summarizes the configurations of the scenarios, taking into account that 156 users, two central offices, three base stations, in addition to 13 UDAPs for WiFi and 20 UDAPs for Zigbee are established. In WiFi, each UDAP functions as an access point, while in Zigbee, each UDAP is an area coordinator, and a central office acts as the general coordinator of the network. In the case of WiFi, the 2400 MHz band is used, as it is openly available for the region, while in the case of Zigbee, it conforms to the Australian standard, which sets the 915 MHz band. Another difference between the two scenarios is the propagation model because Okumura-Hata is adapted to the frequency band that Zigbee operates in this part of Australia, while Two-Ray is coupled to the frequency band in which WiFi operates. The other configurations are also determined based on the technology in use for each scenario.

For the two scenarios to be simulated, many data packets are generated and sent between some nodes to test the performance in the transfer of information. Therefore Table 5 is presented, which indicates the constant bit rate in WiFi technology for chosen nodes; from this table, there is some packets, their corresponding weight, and the sending time interval to obtain; as a result, a total of data sent in the established time from the beginning to the end. Furthermore, It allows to verify that n some nodes queuing will be generated due to the amount of simultaneous incoming and outgoing data; therefore, by applying the first-in, first-out (PEPS or FIFO) method, it will be possible to establish which of the incoming data can be sent out and which are dropped. Similarly, the information to be tested for the Zigbee scenario can be verified in Table 6.

**Figure 8:**
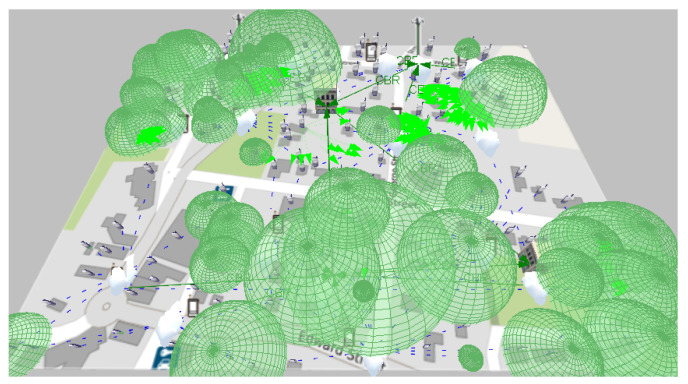
Scenario simulation with 100-m range WiFi technology.

**Figure 9:**
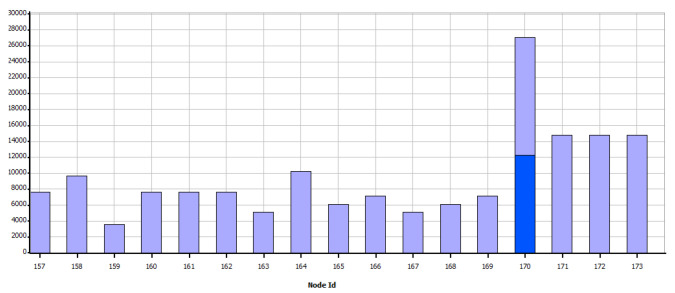
Total data (bytes) sent per node—WiFi.

**Figure 10:**
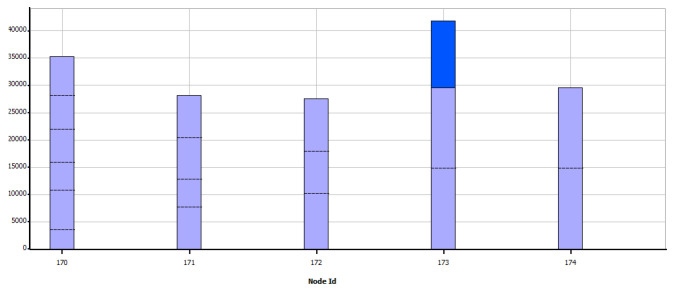
Total data (bytes) received per node—WiFi.

As shown in Figure 8, the simulation shows the sending of data as programmed and visually verifies the communication paths and the radiation patterns or lobes that are generated as the simulation ends so that the results of the network performance can be visualized. In the case of WiFi, the metric shown in Figure 9 is obtained, which shows the data measured in bytes sent by each independent node, according to the programming defined in Table 5. In contrast, as shown in Figure 10 which indicates the data received by each node, it can be verified that of the 18 sets of data sent, 15 are received; which means that within the communication process, three packets were lost, therefore, it is necessary to mention that this result varies according to the amount of traffic and number of packets sent simultaneously between nodes.

**Figure 11:**
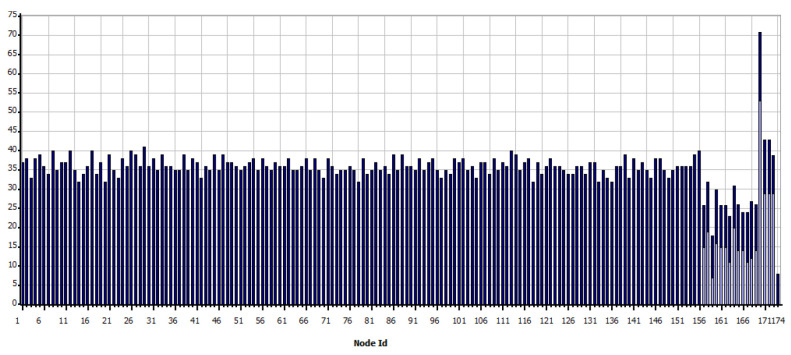
Total packets queued—WiFi.

Figure 11 shows that from the nodes that are sent constant scheduled information, data generated by the interaction and communication hops of the network are also queued, here node 170 stands out, which corresponds to the base station in the southern region of the scenario, which has a more significant amount of data to receive and send.

**Figure 12:**
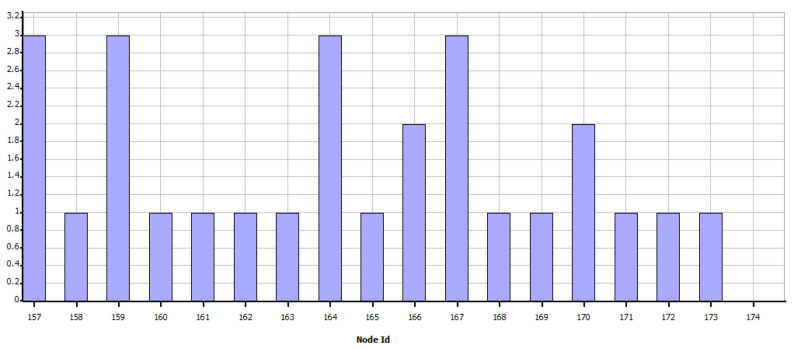
Total hop count for all routes—WiFi.

Therefore, it is clear that nodes that receive more than one packet will generate data queuing according to the order in which they have arrived. Consequently, it is also confirmed that during the establishment time for sending and receiving packets, there is information that will wait the necessary time to reach its destination; however, there are other packets that, having completed the waiting time, will be lost and will not be able to reach their destination. On the other hand, through the Figure 12, it is confirmed that the devices search for a suitable communication route using the reactive AODV (Ad hoc On-Demand Distance Vector Routing) protocol, considering that they can make more than one hop to reach their destination and that the necessary multi hops have been established to meet the information transfer needs.

**Figure 13:**
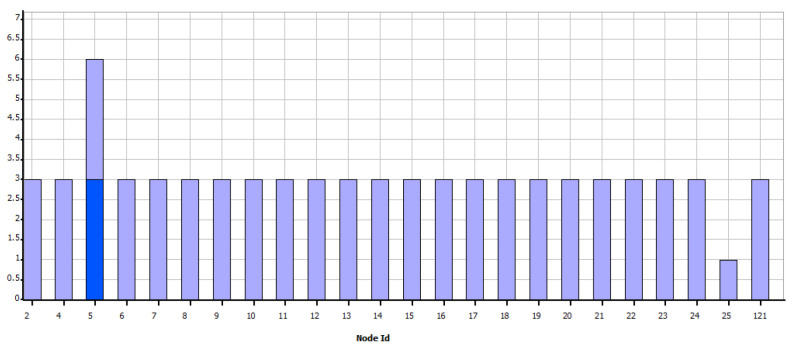
Data sent by node—Zigbee.

In this Zigbee scenario, Figure 13 shows the number of times in which links have been broken in each link. The data rate of the technology limits the time and capacity of packet queuing. In addition, to the saturation of the routes.

**Figure 14:**
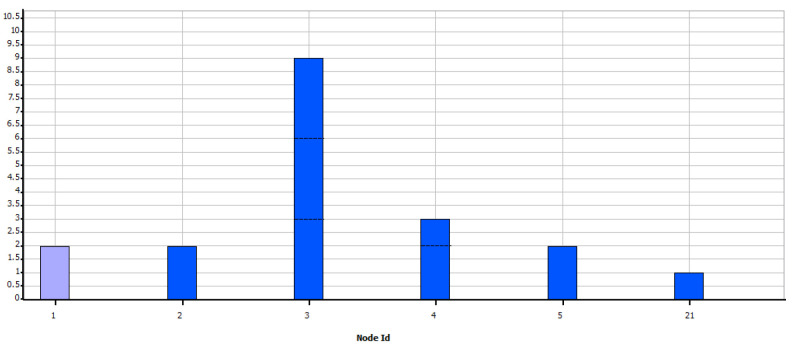
Data received per node—Zigbee.

**Figure 15:**
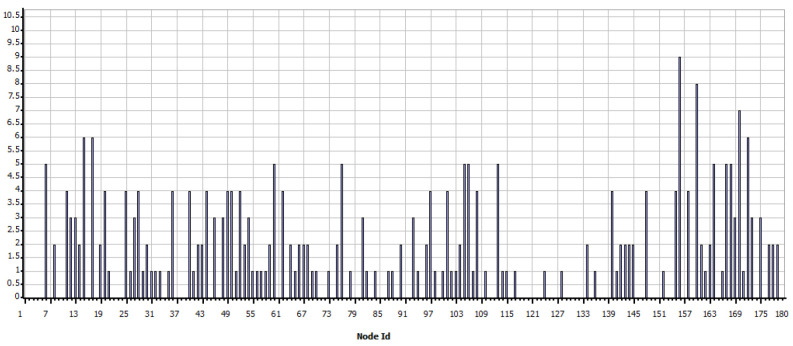
Broken links at each node—Zigbee.

In the case of Zigbee, a different result has been obtained concerning WiFi, while Figure 14 shows the data sent, in Figure 15 shows the data received. From these graphs, it is evident that a large amount of information is lost in the transfer since this could not be kept in a queue or otherwise found saturated paths to their destination.

In addition, it is explained considering that there is a greater sensitivity of the technology to queue data, the type of scenario according to the propagation model, the scheduling of sending data between nodes, and the interference between the same nodes deployed.

Everything that has been developed in this work has been based on the use, in the first instance, of Matlab R2020b to demonstrate the performance of the optimization model, while for the simulation process was made use of the simulator QualNet Developer 5.2, the characteristics of the PC used for the use of the mentioned programs are: Intel Core i5-7200U CPU @ 2.50 GHz 2.70 GHz and 16 GB RAM.

## 6. Conclusions

This study has demonstrated that the model applied to the optimization of advanced metering infrastructure resources is practical. In the proposed case study, it has been possible to minimize the number of concentrators for any admissible value in the respective restrictions. It is essential to mention that it has been possible to establish some scenarios that allow verifying that different feasible solution paths can be obtained to modify the parameters and restrictions.

It can be said that under the tested scenarios and depending on the deployed concentrators, in the worst-case scenario, at most minuscule 28 % of concentrators is reduced.

Hence, it is evident that a good performance can be obtained for any technology depending on its range and capacity. However, it can also be determined that for the proposed case study, from 200 m on wards, the result of the model is invariant, so it can be deduced that there is no better solution for the case study, even if the range in meters of each device or its capacity is increased.

Additionally, it is also established that an adequate calculation of distances between nodes using Haversine’s formula, a preliminary indication of the minimum spanning tree using Prim, and a selection of candidate UDAPs through Greedy SCP is necessary to finally use Dijkstra and obtain the respective routing of the entire network.

According to this, it is interpreted that in the face of capacity and range variations, the model allows establishing an adequate number of UDAPs in the network and a set of link routes that establish the communication relationship within the optimized topology.

The capacity restriction in the UDAPs affects the proposed model because the program has more work if there is more than one UDAP that can group a single SM. However, if there is less conglomeration capacity, the decision time is limited, and therefore the program becomes more efficient and returns optimal results in relatively more minor time.

According to this assertion, it can be said that the higher the capacity value, the longer the decision-making time for the proposed case study. On the other hand, with the topology deployed in Matlab, it has been possible to create geo-referenced scenarios in the QualNet simulator to test the network performance.

## Figures and Tables

**Table 1:** Area network requirements.

Area Network	Data Speed	Coverage Range	Bandwidth	Latency	Traffic
HAN	Low data rate 1–30 kbps	Ten meters	Low	2–15 s	Periodic, 15–60 min
NAN	10–100 kbps	17/5000 Translation results Hundreds of meters	Middle	10 ms a 2 s	Periodic
WAN	High data rate.Hundreds of Mbps a few Gbps	Ten kilometers	Wide	few ms a 1 s	Random

**Table 2:** Wireless technology parameters.

Technology	Standard	Transmission Rate	Distance	Frequency Band
Zigbee	IEEE 802.15.4	256 kbps	Up to 100 m	2.4 GHz (Worldwide), 784 MHz (China), 868 MHz (Europa) 915 MHz (EEUU y Australia)
WiFi	IEEE 802.11b/g/n	1–54 Mbps (b/g) 26–600 Mbps (n)	Up to 100 m (b/g) Up to 200 m (n)	2.4 and 5 GHz (b/g/n)
LoRa	-	Up to 50 kbps	Up to 20 km	433/868/915 MHz
2.5G GPRS/3G UMTS/4G LTE	-	144 kbps (2.5G)/14.4 Mbps (descent) and 5.75 Mbps (ascent)—HSPA—84 Mbps (descent) and 22 Mbps (ascent) en HSPA+/326 Mbps (descent) and 86 Mbps (ascent)—LTE	Up to 5 km	900/1800 MHz
WiMAX	IEEE 802.16	128 Mbps (descent) and 28 Mbps (ascent)	Up to 10 km	433/868/915 MHz

**Table 3:** Notation of model variables.

$X_{u},Y_{u}$	Geographic coordinates of users (Lat., Long.)
$X_{d},Y_{d}$	Geographic coordinates of UDAP (Lat., Long.)
$X_{eb},Y_{eb}$	Geographic coordinates of base stations (Lat., Long.)
$X_{c},Y_{c}$	Head office geographic coordinates (Lat., Long.)
*N*	Number of users
*M*	Number of UDAP
*K*	Number of base stations
*P*	Number of base stations
$X_{T},Y_{T}$	Geographic coordinates of all nodes (Lat., Long.)
*D*	Distance between nodes
*G*	Logical matrix of links between nodes
$D_{max}$	Range in kilometers of the devices
*A*	Compressed matrix of G
rp	Row indicator of the compressed matrix
ci	Column index of the compressed matrix
ai	Compressed array value index
pred	Path predecessor tree function, Dijkstra
nodo	Device Index
path	Tree Path Set
CostR	Cost of each route
Cost	Cumulative vector of costs per route
CostT	Tree Path Adder
CapNodo	Maximum capacity of each node
Coverage	Maximum distance in kilometers of reach of the nodes
*B*	Logical matrix of connections between users and UDAP
tmp	Time vector of users connected to concentrators, Prim
*s*	User count per UDAP
*L*	Matrix of users connected to each UDAP
ind	Matrix of users not connected to UDAP
solC	Resulting transposed logic matrix, Greedy SCP
solL	Optimal UDAP matrix, Greedy SCP
*R*	SolL ordered array without repeating UDAP
$X_{el},Y_{el}$	Geographical coordinates of optimal concentrators (Lat., Long.)
G2	Matrix of weights per link between nodes

**Table 4:** Configuration summary of the scenarios to simulate.

Configuration	WiFi	Zigbee
Land	Geographical coordinates	Geographical coordinates
Simulation time	00:00:30	00:01:15
Channel	2400 MHz	915 MHz
Propagation model	Two-Ray	Okumura-Hata
Routing Protocol	AODV	AODV
Radio Type	802.11b	802.15.4
Potency of transmission	15 dBm	3 dBm
Package reception model	PHY802.11b	PHY 802.15.4
Modulation scheme	-	O-QPSK
Antenna model	Omnidirectional	Omnidirectional
MAC protocol	802.11	802.15.4
Network protocol	IPV4	IPV4
Device type	Generic	Full function devices
Number of nodes	174	181
Access points	13	-
Number of networks	19	1

**Table 5:** Constant bit rate between nodes—WiFi simulation.

Source Node	Destination Node	Start (s)	Final (s)	Items	Pack Size (bytes)	Interval	Total (kbytes)
170	174	1	30	100	512	1	1484,8
172	173	1	30	100	512	1	1484,8
171	173	1	30	100	512	1	1484,8
157	171	10	30	15	512	1	153,6
161	171	1	20	15	512	1	145,92
163	171	5	25	15	512	2	76,8
162	171	1	30	15	512	2	111,36
160	172	1	30	20	512	2	148,48
158	172	1	20	20	512	1	194,56
164	172	5	25	20	512	1	204,8
167	170	10	29	15	512	2	72,96
159	170	15	29	20	512	2	71,68
166	170	1	15	20	512	1	143,36
165	170	5	29	20	512	2	122,88
168	170	5	20	12	512	1	92,16
169	170	15	29	20	512	1	143,36
173	174	1	30	100	512	1	1484,8
170	173	1	25	100	512	1	1228,8

**Table 6:** Constant bit rate between nodes—Zigbee simulation.

Source Node	Destination Node	Start (s)	Final (s)	Items	Pack Size (bytes)	Interval	Total (kbytes)
25	6	1	3	10	512	1	10,24
8	4	3	6	20	512	1	30,72
12	4	6	9	20	512	1	30,72
9	4	9	12	20	512	1	30,72
11	4	12	15	20	512	1	30,72
15	4	15	18	20	512	1	30,72
4	1	18	21	20	512	1	30,72
2	1	21	24	20	512	1	30,72
19	5	24	27	20	512	1	30,72
18	5	27	30	20	512	1	30,72
16	5	30	33	20	512	1	30,72
17	5	33	36	20	512	1	30,72
23	5	36	39	20	512	1	30,72
20	5	39	42	20	512	1	30,72
24	5	42	45	25	512	1	38,4
22	5	45	48	25	512	1	38,4
5	2	48	51	25	512	1	38,4
5	1	51	54	25	512	1	38,4
21	5	54	57	25	512	1	38,4
121	21	57	60	25	512	1	38,4
6	3	60	63	25	512	1	38,4
7	3	63	66	25	512	1	38,4
10	3	66	69	25	512	1	38,4
13	3	69	72	25	512	1	38,4
14	3	72	75	25	512	1	38,4

## Data Availability

Not applicable.
